# A Preliminary Study of Chemically Preserved and High-Moisture Whole Maize (*Zea mays* L.) Usage in Pekin Duck Nutrition: Effect on Growth Performance and Selected Internal Organ Traits

**DOI:** 10.3390/ani11041018

**Published:** 2021-04-04

**Authors:** Bartosz Kierończyk, Mateusz Rawski, Zuzanna Mikołajczak, Roksana Wachowiak, Natalia Homska, Damian Józefiak

**Affiliations:** 1 Department of Animal Nutrition, Faculty of Veterinary Medicine and Animal Science, Poznań University of Life Sciences, Wołyńska 33, 60-637 Poznań, Poland; zuzanna.mikolajczak@up.poznan.pl (Z.M.); wachowiak.roksana@gmail.com (R.W.); damian.jozefiak@up.poznan.pl (D.J.); 2 Laboratory of Inland Fisheries and Aquaculture, Department of Zoology, Faculty of Veterinary Medicine and Animal Science, Poznań University of Life Sciences, Wojska Polskiego 71c, 60-625 Poznań, Poland; mateusz.rawski@up.poznan.pl (M.R.); nhomska@gmail.com (N.H.),

**Keywords:** whole grain, maize, duck, performance, GIT measurements

## Abstract

**Simple Summary:**

Harvested maize grains characterized by high moisture require the use of additional preservation processes to secure against the microbiota. The most commonly used practice is heat-drying, which is energy- and cost-consuming, as well as environmentally unfriendly. Thus, there is a need to omit these harmful techniques and evaluate the usage of wet maize in animal nutrition. The present study aimed to investigate the effect of the chemically preserved, high-moisture whole maize grain addition in Pekin duck nutrition on their growth performance and selected internal organ and gastrointestinal tract measurements and digesta pH values. The results of the experiment clearly showed that there is a possibility to partially replace commonly used heat-dry maize with the preserved whole-grain form without an adverse effect on the birds’ performances and selected organ morphometrical parameters. However, due to surprising changes in the pH values in the gizzard and caeca, further investigations, including microbiota analyses, are recommended.

**Abstract:**

This study aimed to investigate the effect of chemically preserved, high-moisture whole maize grain addition in Pekin duck diets on their growth performance and selected internal organ and gastrointestinal tract measurements and digesta pH values. A total of 300 29-d-old male Pekin ducks were randomly distributed into three dietary treatments using five replicate pens per group and 20 birds per pen. The following treatment groups were applied: CON—basal diet, 5HM—5% of high-moisture, chemically preserved whole maize (HM) inclusion, and 10HM—10% of HM addition. The experiment lasted 21 d. The implementation of 5HM or 10HM did not affect (*p* > 0.05) the growth performance parameters, selected internal organ weights, and the gastrointestinal tract segment weights and lengths. However, significant changes in terms of the gizzard (*p* = 0.005), ileum (*p* = 0.030), and caecal (*p* < 0.001) digesta pH were observed, especially in the case of the 10HM group, which exhibited the greatest increase in pH in the gizzard and caecal digesta and decrease in the ileal digesta pH. The implementation of whole wet maize may be used in waterfowl diets from 29 d of age. Additionally, chemical preservation can efficiently reduce the cost of maize preparation in duck nutrition.

## 1. Introduction

The beneficial impact of whole-grain diets on livestock nutrition is well-documented [1,2,3,4]. In poultry feed, wheat [5], barley [6], maize [7], and sorghum [8] are predominantly used in their whole-grain form. Their positive effects are mainly the improvement of poultry growth performance, particularly the feed intake (FI) and feed conversion ratio (FCR), nutrient and energy utilization, gizzard development, decreased mortality rates, reduced feed production costs, and increased feed mill capacity [9]. In terms of the vegetation conditions in Poland, as well as in many other European countries, from the above-mentioned feed materials, maize is characterized by the highest water content range, between 25–36% during harvesting [10]. Due to this fact, further processing is required to limit mold growth and, consequently, prevent the appearance of a wide spectrum of mycotoxins, such as deoxynivalenol (DON) and zearalenone (ZON) [11]. Thus, the commonly used preservation techniques based on moisture removal, i.e., a moisture content below 13% on a wet weight basis, are implemented. However, high-temperature (80–140 °C) methods pose a risk of negative effects on bird performances through altered starch and protein apparent ileal digestibility [12]. Furthermore, Métayer et al. [13] indicated that the drying process may decrease the feed energy utilization by birds. However, from a practical point of view, the usage of air-dried maize as a whole material in poultry nutrition is the most common, and its processing is linked with additional energy consumption. Nevertheless, mixtures of selected organic acids or their salts are well-known as preservatives. The combination of calcium formate, sodium benzoate, and sodium nitrite efficiently inhibited ZON, fumonisins (FUMs), ochratoxins (OTAs), and DON [14]. Predominantly, acetic, propionic, formic, butyric, and benzoic acids are used to prevent fungal growth [15,16,17]. There are limited data about the usage of chemically preserved maize in poultry nutrition, particularly in that of waterfowl. To date, only Konieczka et al. [10] implemented chemically preserved, high-moisture maize (150 g kg^−1^) in turkey diets, with no detrimental effects on bird growth performance, carcass characteristics, and blood redox status. According to the available literature, due to the usage of the various physical forms of the diet in the present study, the authors assumed the hypothesis that the whole and high-moisture maize added to the duck diets may affect the growth performance parameters, as well as the development of the gastrointestinal tracts (GIT) of birds. Additionally, the favourable economic impact of the usage of high-moisture and chemically preserved whole maize is expected due to the decreased feed material cost.

In addition to the growth performance results, as well as the effects of whole maize on GIT development and physiological reactions, from a practical point of view, the implementation of the chemical preservation of whole maize grains has a beneficial impact on reducing production costs. It is well-documented that the drying process of grains is a highly energy-consuming operation that may expend 10–25% of the national energy use each year [18]. The heat-drying of maize after harvesting consumes 30–50% of all costs of grain preparation in Poland. Generally, the drying of one ton of wet grains consumes approximately 16–23 L of diesel oil or 28 L of liquefied petroleum gas (LPG) [19]. Chakraverty et al. [20] claimed that more than one bn (billion) liters of diesel oil are used in convection dryers, which are environmentally unfriendly due to their promotion of greenhouse gas (GHG) emissions [21]. The demanded prediction of the largest Polish feed mill was estimated to be 90,000 tons of maize annually (considering 300,000 tons of feed annually of which maize constitutes up to 30%), which needs to dry to a moisture content below 13% on a dry matter (DM) basis. This process consumes approximately 16–27 M kWh of energy. According to the latest and average energy cost in Poland (0.55 PLN and 0.12 €), the savings resulting from the use of wet grain may reach up to 2–3.2 M € annually.

To the best of our knowledge, the current study is the first to use chemically preserved whole maize in Pekin duck nutrition. Therefore, this study aimed to investigate the effects of a high-moisture whole maize grain addition in duck diets on their growth performance, selected internal organ parameters and gastrointestinal tract (GIT) measurements, as well as digesta pH values.

## 2. Materials and Methods

### 2.1. Ethics Statement

According to Polish law and the EU directive (no. 2010/63/EU), the experiments conducted within the study did not require the approval of the Local Ethical Committee for Experiments on Animals in Poznań. All procedures complied with the animal experimentation guidance and care of animals under study, and all efforts were made to minimize suffering.

### 2.2. Birds and Housing

In total, 300 29-d-old male Pekin ducks (initial weight—2 kg ± 80 g) were randomly distributed to 3 dietary treatment groups using 5 replicate pens per group and 20 birds per pen as an experimental unit. The birds were kept in floor pens arranged by a completely randomized design in the center of a commercial duck house until 50 d of age. To simulate intensive production conditions, the experimental pens were surrounded by a duck flock composed of birds of the same origin as those used in the experiments. All pens contained 1 bell drinker and 1 feed hopper. The commercial duck house was equipped with artificial, programmable lights, automatic electric heating, and forced ventilation. The temperature inside the building was 30 °C (29 d) at the beginning of the test and was reduced by 2 to 3 °C each week until reaching 24 °C at the end of the trial. The humidity was maintained at 70–75%.

### 2.3. Pekin Duck Diets

The composition of the experimental diets is presented in Table 1. Birds had ad libitum access to water and feed for 21 d (experimental period). The control diet was calculated to meet or exceed the nutrient requirements recommended by the National Research Council (1994) for Pekin ducks and produced in the Piast Pasze feed mill (Lewkowiec, Poland) according to the ISO 9001:2008 procedures. The feed was prepared on a laboratory-scale line and offered to the birds in a pelleted form (3.2 mm in diameter and 70 °C in the pelleting process) in the control diet or pelleted feed with 5% or 10% addition of whole maize (95% of pelleted concentrate with 5% whole, high-moisture maize addition and 90% of pelleted concentrate with 10% of experimental factor). The control diet was offered from 1 to 28 d of age, and after 29 d, maize in the control diet was partially replaced by the addition of 5% or 10% chemically preserved, high-moisture whole maize. Thus, the nutritive value of the experimental diets was diluted due to the various DM content of heat-dried or high-moisture maize incorporation. It should be highlighted that the diets were differed mainly in terms of the metabolizable energy content. The nutritive value of the maize is presented in Table 2. Exogenous enzymes, i.e., phytase (6-phytase; EC 3.1.3.26; 2010 FYT/kg, DSM Nutritional Products Ltd., Heerlen, Netherlands), as well as endo-1,4-beta-xylanase (EC 3.2.1.8; 200 FXU/kg; DSM Nutritional Products Ltd., Heerlen, Netherlands), were added to the diets. The following treatment groups were implemented: control (CON)—basal diet, 5HM—replacement of maize by 5% addition of high-moisture, chemically preserved whole maize, and 10HM—replacement of maize by 10% addition of high-moisture, chemically preserved whole maize.

### 2.4. Data and Sample Collection

Body weight (BW) and feed intake (FI) were measured, as well as the body weight gain (BWG) and the feed conversion ratio (FCR), were calculated. The growth performance variables were obtained at the beginning (29 d) and the end (50 d) of the trial using an analytical scale (NVL5101, OHAUS, Nänikon, Switzerland) with an accuracy of ± 1 g. Ten randomly chosen individual birds per treatment were used as an experimental unit (2 Pekin ducks from each of 5 replicate pens per treatment, *n* = 10), stunned using electric field exposure, sacrificed, and were eviscerated to collect the materials for further analyses. The selected internal organ weight in relation to the BW (% of BW) of the duodenum, jejunum, ileum, caeca, proventriculus, gizzard, liver, pancreas, heart, spleen, and bursa of *Fabricius*, as well as the lengths in relation to the BW (cm/kg BW) of the duodenum, jejunum, ileum, and caeca, were measured. After dissection, the above-mentioned organs were rinsed in sterile water, drained, and weighed using PS 600/C/2—Radwag (Radom, Poland) precision scales, and their length was determined. The jejunum was considered to begin at the end of the duodenum and end at Meckel’s diverticulum. The ileum was defined as the small intestinal segment caudal to Meckel’s diverticulum. The digesta from the gizzard, jejunum, ileum, and caeca were gently squeezed from these segments from 10 individual birds (2 randomly chosen Pekin ducks from each of 5 replicate pens per treatment, *n* = 10). The pH in the digesta was measured (in triplicate) immediately after slaughter using a combined glass and reference electrode (pH 1000 L, VWR International, Leuven, Belgium).

### 2.5. Maize Preservation Process

The whole maize preservation process was prepared based on Konieczka et al. [10]. Briefly, a mixture of propionic (700 g/kg) and formic (250 g/kg) acids, as well as water (44 g/kg), glycol, and glycerin (3 g/kg each), was sprayed on whole maize grains after harvest. The dose was set as 2 kg of a preservative mixture per ton of harvested grains. The process was achieved in an E9400D grain bagger (San Francisco, Córdoba, Argentina).

### 2.6. Statistical Analyses

The experiment had a completely randomized design. All data were tested for normal distributions using the Shapiro–Wilk test. Bartlett’s test was adopted to evaluate the homogeneity of variance. Duncan’s multiple range post hoc test was used to determine the significance of differences between treatment means at the significance level of *p <* 0.05. Due to the occurrence of nonnormally distributed data, Dunn’s test with Benjamini–Hochberg adjustment for multiple comparisons followed by a significant Kruskal–Wallis test was used. Statistical analyses were performed using SAS software (SAS Institute Inc., Cary, NC, USA). In the experiment, the following general model was used:

(1) $$Y_{i}=\mu +\alpha_{i}+\delta_{ij}$$

where *Y_i_* is the observed dependent variable, *μ* is the overall mean, *α_i_* is the effect of high-moisture, chemically preserved whole maize, and *δ_ij_* is the random error.

## 3. Results

The growth performance results are shown in Table 3. No statistically significant differences were observed between treatments in the case of the final BW (*p =* 0.885), BWG (*p =* 0.804), FI (*p =* 0.962), or FCR (*p =* 0.790). Furthermore, there were no effects of implementing high-moisture, chemically preserved whole maize grains on the selected intestinal segment weights and lengths or internal organ weights (*p >* 0.05; Table 4). Except for the liver weight, which was decreased (*p* = 0.004) by both experimental diets. Interestingly, the pH value in the digesta of the gizzard (*p =* 0.005), ileum (*p =* 0.030), and caeca (*p <* 0.001) was significantly affected by whole maize supplementation in the duck diets (Table 5). The strongest impact was visible in the 10HM treatment group, which exhibited an increased pH value in the gizzard, as well as in the caeca. A reduction in the pH value was noted in the case of the ileum digesta. However, the 5HM treatment affected only the caecal environment by increasing the pH of its contents.

## 4. Discussion

The preservation technique of high-moisture maize grains using various agents, such as chemical or biological agents, has been frequently studied [22,23,24,25]. Without preservation, this feed material exhibited an increasing pH level (from 5.0 to 5.9) until 41 h, consequently enhancing the possibility of Enterobacteriaceae proliferation due to the limitation of lactic acid bacteria populations and the reduction in lactic acid production [26,27]. To date, there are limited data that evaluate high-moisture maize in animal nutrition; however, some works utilizing swine [28,29], broiler chickens [30], laying hens [31], turkeys [10], and waterfowl [32] have discussed the effects of high-moisture maize. The usage of high-moisture maize in poultry diets resulted in no detrimental effects on the growth performance, GIT physiological status, and redox status of the blood [10]. This was consistent with the fact that no significant changes in nutritive value were observed in either of the heat-dried or high-moisture maize grains [33]. No variability was observed due to the fact that both, i.e., heat-dried, as well as wet maize diets, were prepared in a pelleted form. Furthermore, the usage of heat-dried maize could negatively impact the digestibility of starch and, therefore, energy production in vitro and in vivo, particularly when a high temperature (130 °C) is implemented [12,34,35]. In terms of waterfowl production (mainly ducks—95%), the usage of whole maize grains or triticale in the bird diet is well-known to be used in the overfeeding stage in fatty liver (*foie gras*) production in France (CIFOG, 2015). However, according to the authors, the results of the present study are the first to evaluate the usage of high-moisture and chemically preserved whole grain maize in duck diets starting at the earlier stage of feeding, i.e., 29 d of age. The addition of 5% or 10% high-moisture, chemically preserved whole maize to the Pekin duck diets did not negatively affect the growth performance results and compared to those of the control group, even when the dilution of experimental diets nutritive value via the usage of high-moisture maize (low DM content) was provided. It was a result of the relatively small changes in terms of crude protein and amino acids contents of the diets between treatments. Additionally, the reduced energetic level was not negatively triggered by the nutrient’s availability. Additionally, the above-mentioned effect is strictly related to the feed intake behavior, which was not disrupted by the inclusion of whole maize grains in the duck diets, as the result of the feed intake showed. Thus, the probability of the lowering of the nutrient’s digestibility was scarce. These results were in accordance with those of Konieczka et al. [10], Lu et al. [36], and Kokoszynski et al. [37]; however, in those trials, no dilution of nutrients was applied. Additionally, some negative changes in BW and average daily gain were observed in mule ducks with whole maize and triticale grain use from 57 to 87 d of age [32]. Furthermore, Singh et al. [38] reported that the addition of whole wheat grains to broiler diets adversely affected the BWG and FI. Therefore, further investigation of the usage of chemically preserved whole maize is needed.

In general, the addition of whole grain in poultry diets resulted in enhanced gizzard development [32,36,39]. Nevertheless, in the present study, generally, no influence of whole maize was observed on the selected internal organs, as well as on the GIT measurements, including those of the gizzard. This was consistent with the results of Arroyo et al. [32], who suggested a four-week duck nutrition period with the inclusion of whole grains as necessary to allow for GIT segment adaptation to the diet. However, the implementation of the whole maize in the ducks’ diets positively affected the liver mass. It is well-documented that the weight of this organ can be related to fat deposition (lipogenesis), which may be, in consequence, a cause of fatty liver syndrome [40]. The physical form of the diet may enhance the activity of the gizzard and further stimulate the bile salts secretion by the liver [41]. Moreover, the reduced metabolizable energy of feed may affect the liver mass through the reducing fat deposition as well [42]. Nonetheless, the additional digestibility test should be provided to make a direct explanation. Surprisingly, the gizzard pH value was increased by the usage of 10% whole and preserved maize to the duck diet. This change in pH could be inferred to as a negative effect on the optimal pepsin activity (pH 1.5) [43] and gizzard development [44], as well as its effect on digestion efficiency (mainly that of protein) [9]. Notwithstanding, the growth performance results showed no negative impact on the nutrient availability in the bird GIT. Moreover, the whole maize addition in the duck diets positively reduced the pH value of the ileal digesta in an inclusion-dependent manner. The pH value as a microbial activity indicator suggests the enhanced proliferation of lactic acid bacteria in this segment; however, the aim of the present study did not include microbial analyses; thus, further evaluation of this effect should be performed. Additionally, the caecal pH value also increased in both experimental treatment groups, contrary to the results of Jankowski et al. [45] and Bjerrum et al. [46]. No effect of whole-grain post-pelleting addition to the broiler diets was observed in Singh et al. [38].

The usage of chemically preserved and high-moisture whole maize in the ducks’ diets has a beneficial role in the reduced cost of feed production. The 5% or 10% addition of this feed material may reduce the feed price by approximately 4.4 or 8.8 € per ton, respectively. The calculation takes into account the price of dried maize (10.03.2021; Eurostat [47]), which was established at the level of 220 € per ton, as well as 132 € per ton for high-moisture maize (reduced cost by 40% due to the lack of drying process). According to the European Commission DG Agri-Poultry Production (Eurostat [48]), the ducks’ production in the EU in 2020 is estimated at the level of 369 thousand tons of meat. Thus, the feed production for this poultry species should be assessed as 1.3 M tons based on the FCR equal to 2.3, as well as of dressing out percentage, i.e., 65%. As the result of the present study, there is calculated that the usage of the preserved and high-moisture whole maize in the ducks’ nutrition may save up to 11,5 M € in the European conditions.

## 5. Conclusions

The present study suggests the possibility of partially including chemically preserved whole, wet maize in duck diets without a negative effect on the growth performance, as well as internal organ and GIT segment lengths and weights in the evaluated period. However, further investigation in terms of the microbial population and the impact of their activity on the pH value in the selected GIT segments, especially in the gizzard, as well as in the caecal digesta, is required. Moreover, due to the limitations in the present study, it is crucial to expand the analyses, particularly to assess the coefficients of the nutrients’ digestibility, as well as prolong the experiment period. Finally, it is indisputable that the usage of chemically preserved maize has a crucial impact on reducing feed costs in duck nutrition.

## Figures and Tables

**Table 1:** Composition of the experimental diets for Pekin ducks fed from 29 to 50 d of age.

Ingredients, g kg^−1^	CON ^1^	5HM ^2^	10HM ^3^
Wheat	329.61	329.61	329.61
Maize	230.01	180.01	130.01
High-moisture maize grain	-	50	100
Wheat middling	210.02	210.02	210.02
Rapeseed cake	77.40	77.40	77.40
Extruded full-fat soya bean	63.80	63.80	63.80
Maize DDGS	50.00	50.00	50.00
Soybean oil	13.00	13.00	13.00
Dry hemoglobulin	4.00	4.00	4.00
Mineral-vitamin premix ^4^	3.00	3.00	3.00
Limestone	11.20	11.20	11.20
NaCl	2.60	2.60	2.60
Monocalcium phosphate	1.20	1.20	1.20
L-Lysine	2.36	2.36	2.36
L-Methionine	1.40	1.40	1.40
L-Threonine	0.4	0.4	0.4
Calculated Nutritive Value, g kg^−1^			
AME_N_ (kcal/kg)	3000	2973	2946
Crude protein	180.0	179.3	178.6
Crude fat	60.0	59.7	59.4
Crude fiber	50.0	49.8	49.6
Crude Ash	50.0	49.9	49.8
Digestible Amino Acid			
Lysine	7.5	7.5	7.5
Methionine + Cystine	7.0	7.0	7.0
Threonine	5.0	5.0	5.0
Tryptophan	1.7	1.7	1.7
Arginine	8.0	8.0	7.9
Histidine	4.0	4.0	4.0
Valine	7.0	7.0	6.9

^1^ CON—control group diet, ^2^ 5HM—5% replacement by high-moisture, chemically preserved whole maize, and ^3^ 10HM—10% replacement by high-moisture, chemically preserved whole maize. ^4^ The following were provided per kilogram of diet: vitamin A, 10,000 IU; cholecalciferol, 3000 IU; vitamin E, 25.0 mg; menadione, 1.62 mg; vitamin B_12_, 0.015 mg; folic acid, 1.04 mg; choline, 600 mg; D-pantothenic acid, 6.53 mg; riboflavin, 4.00 mg; niacin, 32.00 mg; thiamine, 1.20 mg; biotin, 0.075 mg; pyridoxine, 2.50 mg; BHT (E321), 0.14 mg; Mn (MnO_2_), 70 mg; Zn (ZnO), 40 mg; Fe (FeSO_4_), 40 mg; Cu (CuSO_4_), 8.00 mg; I (CaI_2_O_6_), 0.81 mg; and Se (Na_2_SeO_3_), 0.21 mg.

**Table 2:** The chemical composition of chemically preserved and high-moisture whole maize (g kg^−1^ as-is) used in this study.

Chemical Composition, g kg^−1^	Heat-Dried Maize	High-Moisture Maize
Dry matter	864.00	722.00
Crude protein	83.00	69.36
Starch	641.00	535.65
Crude fat	37.00	30.92
Crude fiber	22.00	18.38
Crude ash	12.00	10.03
Calcium	0.40	0.33
Sodium	0.04	0.03
Total phosphorus	2.70	2.26
Lysine	2.48	2.07
Methionine + cysteine	3.50	2.93
Threonine	2.94	2.46

**Table 3:** Effect of partial replacement of maize (CON) by a 5% or 10% addition of high-moisture, chemically preserved whole maize on the growth performance of Pekin ducks (29–50-d period).

Item	Treatment			SEM ^4^	*p*-Value
	CON ^1^	5HM ^2^	10HM ^3^		
Final BW, kg ^5^	4.02	4.01	4.06	0.05	0.885
BWG, kg ^6^	2.03	2.00	2.07	0.04	0.804
FI, kg ^7^	5.83	5.78	5.82	0.08	0.962
FCR, kg:kg ^8^	2.89	2.89	2.82	0.05	0.790

^1^ CON—control group, ^2^ 5HM—5% replacement by high-moisture, chemically preserved whole maize, ^3^ 10HM—10% replacement by high-moisture, chemically preserved whole maize, ^4^ SEM—standard error of the mean, ^5^ BW—body weight, ^6^ BWG—body weight gain, ^7^ FI—feed intake, and ^8^ FCR—feed conversion ratio. Means represent 5 pens of 20 birds each (*n* = 5).

**Table 4:** Effect of partial replacement of maize (CON) by a 5% or 10% addition of high-moisture, chemically preserved whole maize on the selected internal organ and gastrointestinal tract segment weights (% of BW) and lengths (cm/kg BW) of Pekin ducks (50 d of age).

Item	Treatment									SEM ^4^	*p*-Value
	CON ^1^			5HM ^2^			10HM ^3^				
Weight (% of BW ^5^)											
Proventriculus	0.22	±	0.03	0.21	±	0.02	0.20	±	0.02	<0.01	0.107
Gizzard	2.67	±	0.46	2.63	±	0.29	2.81	±	0.38	0.06	0.382
Duodenum	0.40	±	0.05	0.37	±	0.05	0.36	±	0.04	0.01	0.065
Jejunum	0.95	±	0.07	0.92	±	0.13	0.93	±	0.08	0.02	0.718
Ileum	0.87	±	2.43	0.87	±	0.09	0.88	±	0.07	0.01	0.685
Caeca	0.17	±	0.02	0.17	±	0.03	0.16	±	0.03	<0.01	0.134
Heart	0.51	±	0.06	0.51	±	0.06	0.54	±	0.04	0.01	0.211
Pancreas	0.25	±	0.05	0.22	±	0.03	0.23	±	0.02	0.01	0.211
Liver	2.27 ^a^	±	0.37	1.90 ^b^	±	0.20	1.91 ^b^	±	0.16	0.05	0.004
Spleen	0.06	±	0.02	0.06	±	0.01	0.07	±	0.01	<0.01	0.544
Bursa of *Fabricius*	0.09	±	0.03	0.10	±	0.03	0.09	±	0.02	<0.01	0.723
Length (cm/kg BW ^5^)											
Duodenum	10.85	±	0.92	10.37	±	0.93	10.41	±	0.74	0.15	0.223
Jejunum	23.38	±	2.67	21.61	±	2.56	23.96	±	1.95	0.42	0.577
Ileum	23.98	±	3.04	22.02	±	2.26	24.43	±	2.04	0.44	0.685
Caeca	5.75	±	0.44	5.21	±	0.43	5.19	±	0.48	0.08	0.916

^1^ CON—control group, ^2^ 5HM—5% replacement by high-moisture, chemically preserved whole maize, ^3^ 10HM—10% replacement by high-moisture, chemically preserved whole maize, ^4^ SEM—standard error of the mean, and ^5^ BW—body weight. Means represent 10 birds (2 randomly chosen ducks from each replicate pen; *n* = 10).

**Table 5:** Effect of partial replacement of maize (CON) by 5% or 10% of high-moisture, chemically preserved whole maize on the gizzard, jejunal, ileal and caecal content pH values of Pekin ducks (50 d of age).

Item	Treatment									SEM ^4^	*p*-Value
	CON ^1^			5HM ^2^			10HM ^3^				
Gizzard	2.51 ^b^	±	0.45	2.49 ^b^	±	0.68	3.52 ^a^	±	1.17	0.16	0.005
Jejunum	6.30	±	0.20	6.23	±	0.48	6.17	±	0.47	0.07	0.753
Ileum	7.32 ^a^	±	0.30	7.12 ^ab^	±	0.35	6.98 ^b^	±	0.24	0.06	0.030
Caeca	5.74 ^b^	±	0.52	6.43 ^a^	±	0.57	6.64 ^a^	±	0.39	0.10	<0.001

^a,b^—means not sharing a common superscript differ significantly (*p* < 0.05), ^1^ CON—control group, ^2^ 5HM—5% replacement by high-moisture, chemically preserved whole maize, ^3^ 10HM—10% replacement by high-moisture, chemically preserved whole maize, and ^4^ SEM—standard error of the mean. Means represent 10 birds (2 randomly chosen ducks from each replicate pen; *n* = 10).

## Data Availability

The data presented in this study (costs calculations) are openly available on the following website, i.e., European Commission, Cereals statistics [47]; and European Commission DG Agri-Poultry Production [48].
